# A small-molecule screen reveals novel modulators of MeCP2 and X-chromosome inactivation maintenance

**DOI:** 10.1186/s11689-020-09332-3

**Published:** 2020-11-10

**Authors:** Hyeong-Min Lee, M. Bram Kuijer, Nerea Ruiz Blanes, Ellen P. Clark, Megumi Aita, Lorena Galiano Arjona, Agnieszka Kokot, Noah Sciaky, Jeremy M. Simon, Sanchita Bhatnagar, Benjamin D. Philpot, Andrea Cerase

**Affiliations:** 1 Department of Cell Biology & Physiology, University of North Carolina School of Medicine, Chapel Hill, NC USA; 2 UNC Neuroscience Center, Carolina Institute for Developmental Disabilities, University of North Carolina School of Medicine, Chapel Hill, NC USA; 3 Current Address: High-Throughput Bioscience Center, Chemical Biology & Therapeutics, St. Jude Children’s Research Hospital, Memphis, TN USA; 4 Blizard Institute, Queen Mary University of London, London, UK; 5 Department of Biochemistry and Molecular Genetics, Department of Neuroscience, University of Virginia School of Medicine, Charlottesville, VA USA; 6 Department of Pharmacology, University of North Carolina School of Medicine, Chapel Hill, NC USA; 7 Department of Genetics, University of North Carolina School of Medicine, Chapel Hill, NC USA

**Keywords:** Rett syndrome, MeCP2, X-chromosome inactivation, AG490, Janus Kinase, Janus Kinase inhibitors, JAK/STAT, PI3K/ATK pathways

## Abstract

**Background:**

Rett syndrome (RTT) is a neurodevelopmental disorder caused by mutations in the X-linked methyl-CpG binding protein 2 (*MeCP2*) gene. While *MeCP2* mutations are lethal in most males, females survive birth but show severe neurological defects. Because X-chromosome inactivation (XCI) is a random process, approximately 50% of the cells silence the wild-type (WT) copy of the *MeCP2* gene. Thus, reactivating the silent WT copy of *MeCP2* could provide therapeutic intervention for RTT.

**Methods:**

Toward this goal, we screened ~ 28,000 small-molecule compounds from several libraries using a MeCP2-luciferase reporter cell line and cortical neurons from a MeCP2-EGFP mouse model. We used gain/increase of luminescence or fluorescence as a readout of MeCP2 reactivation and tested the efficacy of these drugs under different drug regimens, conditions, and cellular contexts.

**Results:**

We identified inhibitors of the JAK/STAT pathway as XCI-reactivating agents, both by in vitro and ex vivo assays. In particular, we show that AG-490, a Janus Kinase 2 (JAK2) kinase inhibitor, and Jaki, a pan JAK/STAT inhibitor, are capable of reactivating MeCP2 from the inactive X chromosome, in different cellular contexts.

**Conclusions:**

Our results suggest that inhibition of the JAK/STAT pathway is a new potential pathway to reinstate *MeCP2* gene expression as an efficient RTT treatment.

**Supplementary Information:**

The online version contains supplementary material available at 10.1186/s11689-020-09332-3.

## Introduction

Rett syndrome (RTT) is a severe neurodevelopmental disorder often classified as an autism spectrum disorder (ASD). About 90% of reported cases of individuals with RTT inherit de novo mutations of the methyl-CpG-binding protein 2 (*MeCP2*) gene [1]. The detailed mechanism of how *MeCP2* mutations lead to RTT is largely unknown; however, mutations in *MeCP2* are associated with defects in neuronal development and synapse formation [2, 3]. A deficiency of *MeCP2* leads to immature synaptic development in the cortex [4–6] and disruption of the metabolism of brain cholesterol, resulting in abnormal neuronal function [7, 8]. Mutations affecting the nuclear localization signal region of *MeCP2* or early truncating mutations are responsible for a severe phenotype in comparison to missense mutations, whereas C-terminal mutations are associated with milder phenotypes [9, 10]. As *MeCP2* is also expressed in glia, dysfunction in glial cells could also be involved in the pathogenesis of RTT [11].

The clinical symptoms in individuals with RTT include breathing abnormalities, loss of speech, motor impairments, loss of purposeful hand movements, and repetitive stereotypical movements [12, 13]. RTT individuals must receive lifelong care because there is currently no cure, although multiple approaches have been used to provide symptomatic relief. For example, antiepileptic drugs provide symptomatic relief for many of the ~ 60% of RTT individuals affected by seizures [14, 15]. Breathing irregularities and sleep problems in RTT individuals can also be symptomatically treated [16–19]. Other treatment options include physical therapy, speech therapy, occupational therapy, and psychosocial support for families. Management of these conditions can substantially improve the quality of life in individuals with RTT [20].

The *MeCP2* gene is located on the X chromosome in both mice and humans. The gene undergoes XCI in females [21], acquiring multiple repressive DNA and histone modifications [22–29]. As XCI is a random process, roughly 50% of each inherited X chromosome is expressed. Consequently, in the case of an X-linked mutation, ~ 50% of wild-type (WT) and mutated X chromosomes are silenced [21]. This silent WT copy on the inactive X chromosome (Xi) represents an important backup that can be potentially unlocked to produce WT protein.

The reversal of XCI, or better yet the specific reversal of *MeCP2* inactivation, offers a promising treatment opportunity for RTT and has been attempted by several groups [30–34]. The feasibility of this approach was first demonstrated by the Green lab, who demonstrated that pharmacologically targeting XCI factors such as ACVR1 and PDPK1 can reactivate *MeCP2* [30]. Subsequently, a small-molecule screen performed by the Lee lab has shown that VX680, an inhibitor of Aurora kinases (AurK), reactivates *MeCP2* if primed by 5-Aza-dC-mediated DNA demethylation [31]. Another study from the Bedalov lab showed that targeting BMP/TGF-beta signaling holds potential for reactivating *MeCP2* on the Xi [33]. And recently, central nervous system co-administration of GSK650394 (SGK1 inhibitor) and LDN193189 (ACVR1 inhibitor) was shown to reactivate *MeCP2* and reverse RTT phenotypes in a mouse model [32]. Collectively, these studies demonstrate the promise of reactivating *MeCP2* by disrupting XCI. However, all these approaches have challenges that still need to be overcome. For example, potential pharmacological treatments (1) can produce cytotoxicity in neuronal cells when administered at higher concentrations [35]; (2) have targets that are barely expressed in neurons, as in the case of AurkA/B [31] (brainrnaseq.org and proteinatlas.com); (3) are not yet shown to reactivate *MeCP2* in neurons [33]; and/or (4) have to be assessed for their ability to produce broad CNS bioavailability with peripheral administration [32]. Until these challenges can be overcome and a treatment for RTT is available, there is an urgent need to continue to identify new *MeCP2* activators and to optimize these and previously identified *MeCP2* activators for safety and efficacy.

Here we employed a phenotypic screen (~ 28,000 compounds) to identify novel compounds that can effectively reactivate the *MeCP2* gene. We found that two JAK/STAT pathway inhibitors, AG490 and Jaki, could reproducibly reactivate *MeCP2*. While the mechanism, efficacy, and safety of targeting this pathway must be thoroughly investigated, our results suggest that JAK-STAT inhibitors offer an additional approach that needs to be explored for the possible treatment of RTT and other X-linked disorders.

## Material and methods

### Animals

All animals were maintained and used according to the Institutional Animal Care and Use Committees of the University of North Carolina at Chapel Hill. Mice were maintained on a 12:12 h light:dark cycle and given ad libitum access to food and water. To produce embryonic cortical neurons for screening, wild-type (WT) female mice were crossed with male mice carrying EGFP-tagged *MeCP2* provided by Adrian Bird at the University of Edinburgh. At 14.5 days post-conception (E14.5), females were terminated to obtain embryos. All genotypes of the offspring were validated by PCR of DNA collected from tail biopsy.

### Chemical libraries

Individual compounds in the chemical libraries were pre-dissolved in DMSO at a concentration of 10 mM/compound. The drug plates for screening were prepared at a final concentration of 1 μM in culture medium. 0.1% DMSO vehicle controls were utilized for screening and all validating experiments. Fresh compounds, AG490 and Jaki, were purchased from Selleckchem and Cayman chemicals, respectively, for compound validation. The full chemical libraries we screened are listed in supplementary Table 1.

### Cell culture and drug treatment

#### Neuron cultures

Cortical neuron cultures were performed by following a previously established protocol [36]. Briefly, neurons were isolated from EGFP reporter mice carrying EGFP-tagged *MeCP2* (*MeCP2*^*EGFP/+*^) or WT mice at E14.5 and allowed to grow for 7 days in vitro (DIV). Neurons were seeded at 20,000 cells per well in 384-well plates and maintained in Neurobasal media supplemented with B27, penicillin/streptomycin, and Glutamax (Invitrogen). At DIV 7, small molecules at a final concentration of 1 μM were added for 3 days. Cortical neurons were also plated at 1 × 10^6^ cells per well in a 6-well plate for western blot analysis. Ex vivo cortical neurons were treated with AG490 (1 and 3 μM) or Jaki (1 and 3 μM) twice for 3 days each.

#### Fibroblast cultures

Mouse tail fibroblast cells carrying an inactive or active *MeCP2*-luciferase reporter (clones Xi8 and Xa3, respectively [33]) were cultured and maintained in DMEM supplemented with 10% FBS and penicillin/streptomycin. Fibroblast cells were plated at 20,000 cells per well in 96-well plates 1 day before treatment. The following day, small molecules (compound libraries) at a final concentration of 1 μM or 5-Aza (10 μM as a control) were added to the cells. The cells were treated for 3 days and tested by means of luminescence assays in a high-throughput manner. For compound validation, the fibroblast cells were treated with AG490 (1 and 3 μM), Jaki (1 and 3 μM), or 5-Aza (10 μM), respectively, for 3 days each or twice for 2 days.

#### THX88 and C2C12 cultures

THX88 is a hamster cell line, containing a single human X chromosome in the inactive state (Xi) [37]. THX88 and C2C12, a fully differentiated female myoblast line [38], were maintained in DMEM supplemented with 10% FBS and penicillin/streptomycin. One day after plating the cells in 6 or 4 multi-well plates (150,000 cells/well), the cells were treated with 3 μM of AG490 or 1 µM Jaki or 10 μM of 5-Aza for 4 days.

#### H4SV cultures

H4SV mouse fibroblast cells were cultured and maintained in DMEM supplemented with 10% FBS and penicillin/streptomycin. In brief, 300,000 cells were seeded into each well of a 6-well plate and cultured 24 h before any treatment. Cells were further treated with AG490 or Jaki during HAT selection for up to 12 days. Surviving cells were counted and measured for GFP reactivation by qPCR (see below) [30, 32].

### Fluorescence immunocytochemistry and high-content imaging

Immunocytochemistry and high-content imaging were performed as previously described [36, 39], using rabbit anti-GFP primary (1:1000, Novus Biologicals) and goat anti-rabbit AlexaFluor-488 secondary (1:500, Invitrogen) antibodies. Hoechst staining was simultaneously performed to visualize and count nuclei. Briefly, images of the immunofluorescence-processed neurons stained for Hoechst and AlexaFluor-488 were analyzed using a BD Pathway 855 high-content imaging fluorescence microscope. MeCP2 reactivation was detected by means of fluorescence intensity in drug-treated cells (at single cell level) vs vehicle control. We defined as potentially active drugs compounds eliciting a consistent increase in the mean EGFP fluorescence, across quadruplicate wells and minimal or no cytotoxicity measured by Hoechst-stained nuclear structure and cell viability. All images were taken by BD Pathway 855, a high-content imager, and processed by CellProfiler [40] to analyze the number of positive GFP cells.

### Luminescence high-throughput screen

Luminescence-based high-throughput screen was performed using mouse fibroblast cells carrying an inactive *MeCP2*-luciferase reporter (Xi8). About 20,000 cells per well were plated into 96-well plates 1 day before compound treatment (*n* = 3 per 1 μM of compounds). Three days after the small-molecule treatment, the cells were rinsed with 1x PBS, and Bright-Glo (Promega) (mixture of substrate and lysis buffer) was directly added to cells in 96-well plates. Fifteen minutes after allowing cell lysis and equilibrating the cell plates in the dark at room temperature, a MicroBeta Trilux (Perkin Elmer) microplate reader was used to measure luminescence from the reactivated MeCP2-luciferase. 5-Aza was used as a positive control [33], and a twofold or more change in relative luciferase unit was considered an active compound (see also Supplementary Table 2).

### Western blot analysis

Western blot analysis was performed to validate the reactivation of MeCP2 protein levels. Six days after small-molecule treatment (2× 3-day treatment), neurons in 6-well plates were harvested to extract total proteins using RIPA buffer. Bradford assays were used to determine protein amount, and 30 μg of total protein was resolved in SDS-PAGE to analyze changes in MeCP2-EGFP protein levels. Primary antibodies were rabbit anti-GFP (1:1000, Novus Biological) and mouse anti-actin (1:5000, Sigma). Secondary antibodies were HRP-conjugated anti-rabbit (1:1000, Vector lab) and anti-mouse (1:1000, Vector Lab). Chemiluminescence produced by ECL substrate (BioRad) was detected by an Amersham Imager 600 (GE Life Sciences).

### RT-PCR and qPCR

RT-PCR and quantitative real-time PCR (qPCR) were performed using previously reported protocols [39]. Total RNA was extracted by using Direct-zol RNA kit and following the manufacturer’s protocol (Zymo Research). cDNA pool was synthesized by qScript cDNA Supermix (Quantabio) using 2 μg of total RNA. cDNA from clone Xi8 and Xa3 RNA was further used for qPCR to analyze reactivated mouse *MeCP2*. qPCR was performed using SsoAdvanced Universal SYBR green Supermix (BioRad). The primer sets were *β-actin* (forward, 5′-AGAGCTACGAGCTGCCTGAC-3′; reverse, 5′-AGCACTGTGTTGGCGTACAG-3), mouse *MeCP2* (forward, 5′-CATACATAGGTCCCCGGTCA-3′; reverse, 5′-CAGGCAAAGCAGAAACATCA-3′), mouse *Xist* (forward 5′-GGTTCTCTCTCCAGAAGCTAGGAAAG-3′; reverse, 5′-GGTAGATGGCATTGTGTATTATATGG-3′), and mouse *Rnf12* (forward, 5′-CTTGGATCGGGAAGAGGCTT-3′; reverse, 5′-TTCACCTGGGGTGCCCAGCA-3′). qPCR was performed using QuantStudio thermocycler (Thermo Fisher) with the following conditions: 95 °C for 5 min and followed by 40 cycles of 95 °C 10 s, 60 °C 1 min. Relative quantity (RQ) was determined after normalized ΔΔC*t* was calculated.

#### Experiments in the Xi-containing line

RNA was extracted using a kit from Qiagen (RNAesy) and subjected to DNAse treatment (Turbo DNAse, Ambion) followed by reverse transcriptase and qRT-PCT analysis (SuperScript III, Thermo; BioRad SYBR green). Primers used were hMECP2–nF: GGCAAGCATGAGCCCGTGCAGCCA, hMECP2–nR: GCCGGGGCGGAGCCTGACCCTTCT; humanXISTF: TGGAGGGAAACAGTATACCCC, humanXISTR: ATTTATGTTGGTTCTTGTGCCC; human Rnf12, RLIMEx1F: ATTGGAGGCGGGCTTGAG, RLIMEx2R: GCAGACTGATCACCACTTCC; hamsterGapdhF: GAGACGCAATGGTGAAGGTC, hamsterGapdhR: GCCTTGACTGTGCCTTTGAA.

Mouse primers used for testing gene reactivation in the HAT selection medium were Mecp2 F: CATGGTAGCTGGGATGTTAGG; Mecp2 R: GCAATCAATTCTACTTTAGAGCG; hprt F: AAGCTTGCTGGTGAAAAGGA; and Hprt R: TTGCGCTCATCTTAGGCTTT. Relative quantity (RQ) was determined after normalized ΔΔC*t* was calculated.

### Statistical analysis

Significant (*p* < 0.05) changes in the MeCP2-EGFP, MeCP2-luciferase levels, and up- or downregulation of tested genes were determined by one-way ANOVA with Dunnett’s multiple comparison test or Student’s *t* tests*.*

## Results

### AG490 is a potential reactivator of X-linked genes

**Fig. 1:**
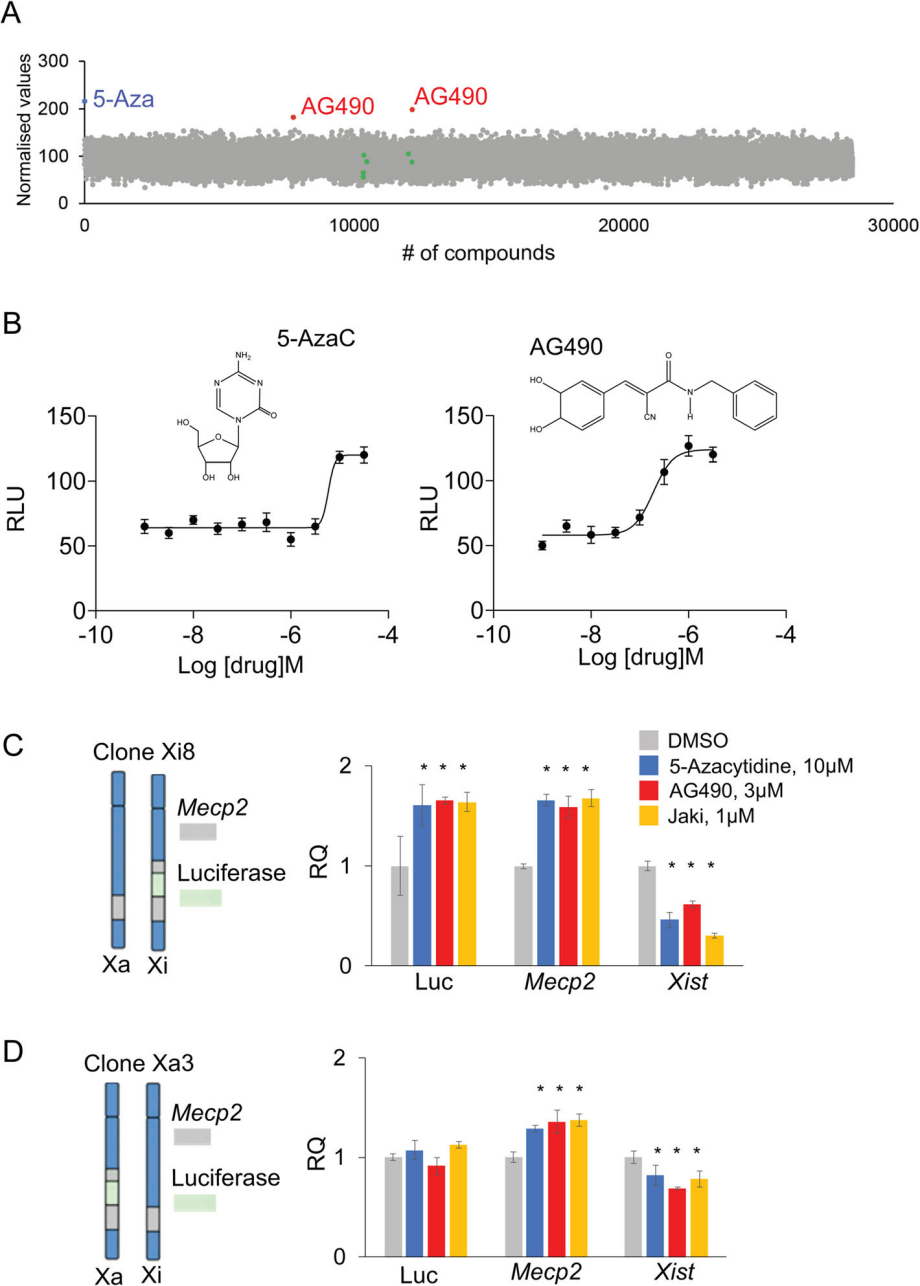
Luminescence-based screen identifies AG490 and Jaki as potential reactivators of genes on the inactive X chromosome. **a** Dot plot indicating the percentage of luciferase activation per drug using the Xi8 fibroblast cell line reporter for MeCP2 activation over the control (normalized values; see the “Material and methods” section). In red, we show AG490, in blue 5-Aza, and in green compounds previously reported to reactivate MeCP2 using different protocols (LY294002, OSU-03012, MLN8237, BX-912, K02288, VX-680; see the “Introduction” and the “Results” sections for more details). **b** Top: Chemical structure of 5-azacytidine (5-Aza), a known reactivator of MeCP2, and AG490. Bottom: Dose response of 3-day treatment of 5-Aza or AG490 in Xi8 fibroblast cells (*n* = 3, one representative graph shown here from three independent assays). **c** Left: Xi8 clone showing *MeCP2* and *MeCP2-luciferase* genes on the inactive chromosome. Right: qRT-PCR data showing the effect of the indicated drug treatments on luciferase (Luc), *Mecp2*, and *Xist* mRNA levels; mean (± SEM) are shown, *statistical significance, *p* ≤ 0.05 (*n* = 3 per compounds, the average of three biological replicates are shown). **d** Left: Xa3 clone showing *MeCP2* and *MeCP2-luciferase* genes on the active chromosome, respectively. Right: qRT-PCR data showing the effect of the indicated drug treatments on luciferase (Luc), *Mecp2*, and *Xist* mRNA levels; mean (± SEM) are shown, *statistical significance, *p* ≤ 0.05 (*n* = 3 per compounds, the average of three biological replicates are shown)

To identify compounds that are capable of reactivating the inactive copy of *MeCP2*, we employed high-throughput, high-content screens using multiple chemical libraries. We screened over 28,000 compounds in two different cell-based models simultaneously. First, we tested our libraries using transformed mouse tail fibroblast cells carrying a MeCP2-luciferase reporter on the inactive X chromosome (clone Xi8) [33]. Luminescence-based screens were performed in a 96-well plate format. Using this approach, we identified AG490 (Fig. 1a, Suppl. Fig. 1A and Table S2) as a potential *MeCP2*-reactivating agent. We used 5-azacytidine (5-Aza), a known XCI-reactivating agent, as a positive control [33]. The EC_50_ of 5-Aza and AG490 was 5.88 μM and 0.19 μM, respectively, in our cell-based screening model, and their efficacy (i.e., level of MeCP2 reactivation) was comparable (*E*_max_ = 120~125 RLU; Fig. 1b). The identified MeCP2-reactivating compounds were further validated using western blot analysis, RT-PCR, and quantitative real-time PCR in different cellular models (see below). We also tested previously published MeCP2 reactivation compounds (LY294002, OSU-03012, BX912 [30]; K02288 [32]; MNL8237, VX680 [31]; see also the “Introduction” section). These compounds failed to reactivate MeCP2 under our experimental conditions (Fig. 1a, green dots).

We further tested the small-molecule libraries in primary cortical neurons generated at embryonic day 14.5 from mice carrying a previously established MeCP2-EGFP reporter [41], with the caveat that the sensitivity of this assay was reduced given that we had to detect increases above a much higher basal level of MeCP2 expression. Although AG490 was identified as a potential activator in the fibroblast cells, the neuronal screen identified no small molecules at the tested concentrations that could consistently reactivate MeCP2 above detection limits in primary neurons (see the “Material and methods” section). Thus, we explored additional drug regimens in an attempt to measure MeCP2 activation with our potential active compounds (see below).

### JAK/STAT inhibitors can reactivate genes on the inactive X chromosome

To validate our primary hit and to explore its possible mechanism of action, we tested two Janus kinase inhibitors (AG490 and Jaki), for their ability to reactivate MeCP2 and Xi in neurons and independent cell lines. AG490 is a Janus Kinase 2 (JAK2) inhibitor used for glioma therapy [42], but it also has inhibitory activity toward the epidermal growth factor receptor (EGFR) mediating PI3K/AKT signaling [43]. Jaki is a *pan* JAK/STAT inhibitor [44]. Furthermore, it has been previously suggested that Jaki can upregulate *MeCP2* in mouse embryonic stem cells [45]. Thus, we used quantitative PCR (qRT-PCR) to test how different doses and time courses of AG490 affected *MeCP2* reversal in comparison with 5-Aza (Fig. 1c, d, and Suppl. Fig. 1B).

First, we tested whether AG490 and Jaki treatments affect the mRNA expression of *MeCP2* from the inactive and active X chromosome (Xi/Xa) in two clonal cell lines. Clones Xi8 and Xa3 are mouse fibroblast lines carrying a *MeCP2*-luciferase reporter on either the inactive X (Xi) or active X (Xa) chromosome, respectively (Fig. 1b, c). We found that AG490 and Jaki (and 5-Aza-positive control) treatments increased the expression of MeCP2 and the luciferase reporter and downregulated *Xist* in the Xi8 clone (Fig. 1b). We similarly found that AG490, Jaki, and 5-Aza increased *MeCP2* and decreased *Xist* levels in the Xa3 clone, without affecting luciferase levels (Fig. 1c). Taken together, these data suggest that the increase in *MeCP2* expression is driven from the Xi rather than the Xa.

We next tested the effect of different AG490 concentrations and time courses on X-linked gene regulation (see also the “Material and methods” section). We report that a single treatment of AG490 (3 μM) is capable of reactivating *MeCP2* in the fibroblast cells without altering RNA expression of *Xist* or *Rnf12*, a strong Xist activator (Fig. S1B, Left). In double-dose treatments, we observed a similar upregulation of *MeCP2* (and its luciferase reporter), and a stronger downregulation of *Xist* and *Rnf12* (Fig. S1B, see the “Material and methods” section). Differences in the treatment regimen can potentially explain the lack of effect of AG490 treatment in the cortical neuronal screen (i.e., a stronger *Xist/Rnf12* downregulation is needed for XCI reversal in neurons, see also the “Discussion” section). Overall, these data support that AG490 and Jaki are potential reactivating agents for X-linked genes on the inactive X chromosome.

We also tested a combination of 5-Aza and AG490 on the Xi8 clone. Surprisingly, the combination of these drugs does not have a synergistic effect on MeCP2 overall reactivation (Fig. S2A, S2B). This observation suggests that MeCP2 reactivation elicited by these drugs might be driven through a pathway or mechanism that may also modulate DNA demethylation.

We next examined whether Xist downregulation might be due to drug-induced, Xist detachment from the Xi. To this end, we performed Xist RNA-FISH on fully differentiated female cell lines (C2C12) treated for 4 days with AG490/Jaki (Fig. S2C)**.** Upon treatment, we did not observe any major change in Xist localization or abundance on the Xi, suggesting that Xist downregulation is likely happening at a transcriptional level.

### AG490 effectively upregulates MeCP2 in cultured cortical neurons and additional cellular contexts

**Fig. 2:**
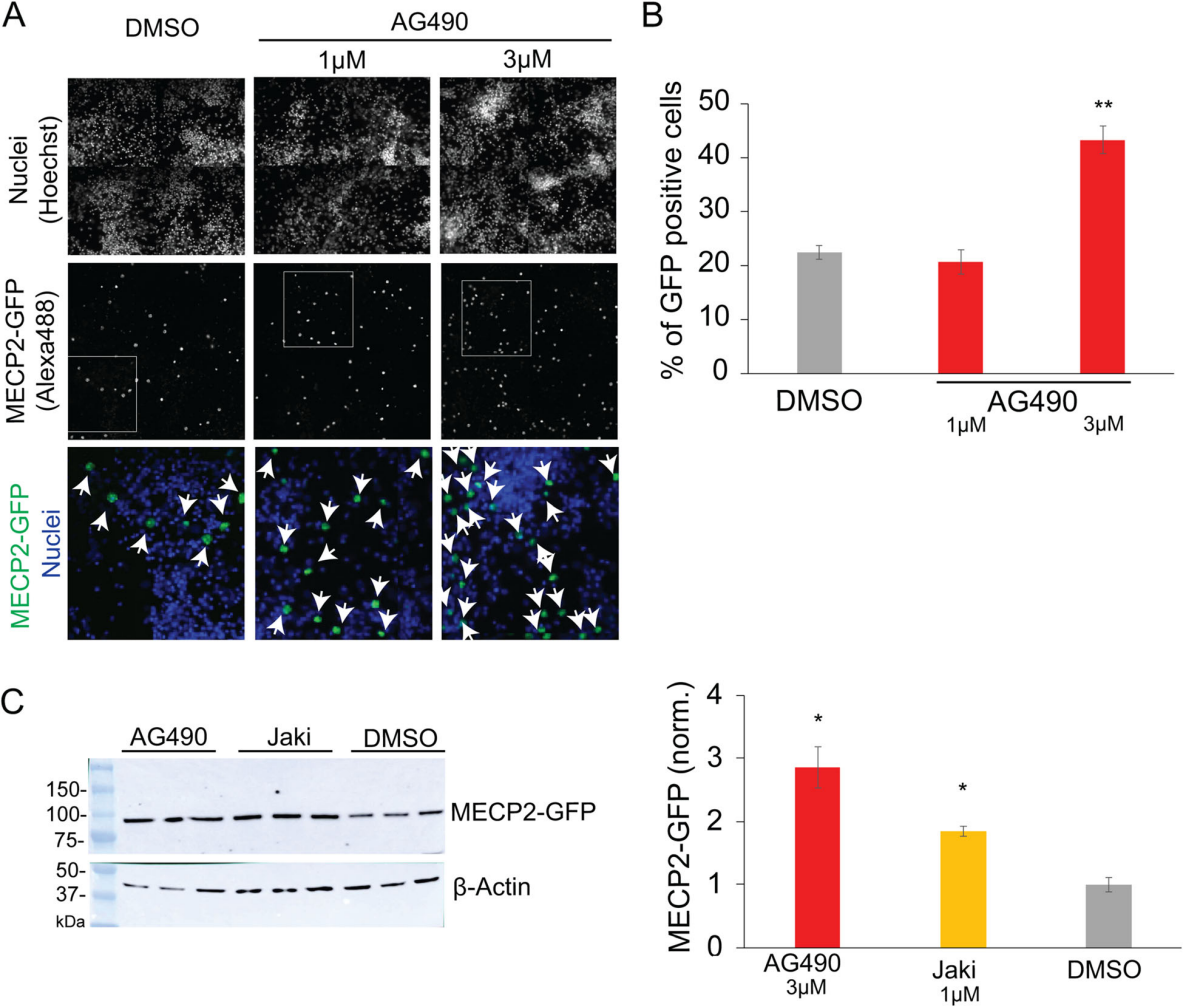
AG490 effectively upregulates MeCP2 in the cultured cortical neurons. **a** Representative fluorescent immunostaining images of cortical neurons after treating with AG490 or DMSO vehicle twice for 3 days each. The top row shows labeling of nuclei, and the middle row shows GFP labeling (of MeCP2-GFP). The bottom row shows the zoom-in of nuclear and MeCP2-GFP labeling from the boxed area depicted in the middle row. Arrows indicate GFP-positive cells. **b** Quantitative analysis of the number of GFP-positive cells (**p ≤ 0.01) (*n* = 4 per dose, the average of three biological replicates are shown). **c** GFP western blot analysis (left) and WB quantification (right) show MeCP2 upregulation in AG490- and Jaki-treated cells, *statistical significance, *p* ≤ 0.05 (*n* = 3 per compounds, the average of two biological replicates are shown)

Given the improved knockdown of *Xist* with a double treatment of AG490, we used this approach to re-explore its ability to activate *MeCP2* in ex vivo neurons. These cultured neurons display a mosaic expression of MeCP2 and MeCP2-EGFP, in which we detect a basal expression of MeCP2-EGFP above a set threshold in 20–25% of cells (DMSO vehicle-treated cell in Fig. 2a, b, and Suppl. Fig. 1C). We found that two treatments of AG490 (3 μM) successfully upregulated MeCP2 in the cultured primary cortical neurons, such that ~ 50% of cells now expressed MeCP2 above threshold (Fig. 2a, b, and Suppl. Fig. 1C). We performed western blot analysis to verify that AG490 upregulated MeCP2-EGFP at the protein level in primary neurons (Fig. 2c). We also found that Jaki (1 and 3 μM) could increase MeCP2-EGFP expression at the tested concentrations (Fig. 2c and Suppl. Fig. 1C).

**Fig. 3:**
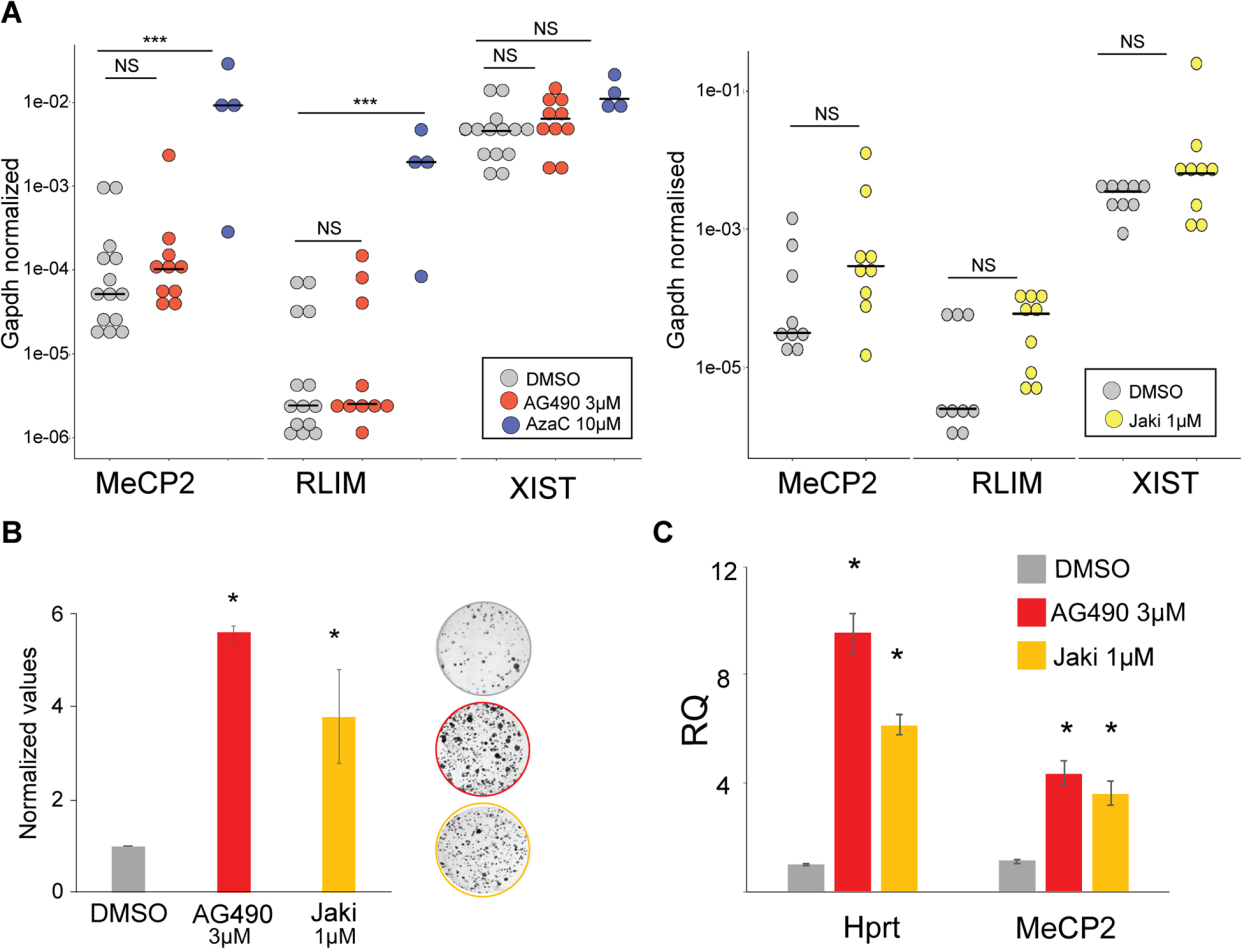
AG490 upregulates MeCP2 in different cellular contexts. **a** qRT-PCR analysis of AG490 treatments in a humanized Xi-containing line (THX88) is shown. Dots indicate individual experimental points, samples are color-coded (see legend), and concentrations are indicated. ***Statistical significance, *p* ≤ 0.001, one-way ANOVA. The experiments were done using 2 (5-Aza), 3 (Jaki), and 4 (AG490) biological replicates, using 2 to 3 technical replicates per experiment. Horizontal lines displayed the median of the samples. Gapdh was used as an internal normalization control. **b** Survival analysis of H4SV cells in HAT-selecting medium in the presence or absence of Xi-reactivating compounds. Fold changes (FC, A.U) of the total number of colonies for control (DMSO) and Xi-reactivating drugs are shown. Bars indicate the standard deviation (SD). *Statistical significance, *p* ≤ 0.05. The average of three biological replicates are shown. Representative plates are shown below. **c** qRT-PCR data showing the effect of the indicated drug treatments on *Mecp2*, Hprt, and *Xist* mRNA levels; mean (± SD) are shown, *statistical significance, *p* ≤ 0.05 (*n* = 3 per compounds, the average of three biological replicates are shown)

To further test the ability of AG490 and Jaki to reactivate *MeCP2*, we tested the compounds in additional cellular contexts. We found that a 4-day treatment of AG490 induces a small, non-significant reactivation of the human *MeCP2* gene in a chimeric cell line carrying a single human inactive X (Xi) chromosome in a hamster background [37] (Fig. 3a). Jaki treatments on the other hand increased MeCP2 reactivation more robustly than AG490, although the extent of MeCP2 reactivation did not reach statistical significance due to inter-experiment variation (Fig. 3a). In this chimeric cell line, 5-Aza significantly reactivated the human *MeCP2* and RNF12 genes to varying degrees, without significantly altering *XIST* levels.

We also tested AG490 and Jaki in a mouse fibroblast cell line, H4SV, in order to provide additional insights into cell-type bias against drug responses. H4SV cells carry a deletion of the hypoxanthine phosphoribosyl transferase (*Hprt*) on the active X and undergo cell death during hypoxanthine-aminopterin-thymidine (HAT) selection (Fig. 3b). AG490 treatment for 7 days allowed the cells to increase their survival rate during HAT selection for 12 days (Fig. 3b), suggesting that AG490 reactivates the *Hprt* gene on inactive X chromosome and providing additional orthogonal evidence that AG490 likely increases MeCP2 expression by activation of the Xi. Hprt and MeCP2 reactivation were also confirmed at the RNA level by qRT-PCR (Fig. 3c).

## Discussion

Previous studies demonstrating the ability of genetic manipulations or small molecules to reactivate *MeCP2* on the inactive X chromosome have shown the promise of this approach as a potential RTT treatment [30–33], yet none of the existing drugs has been applied to preclinical or clinical trials. There is thus a need to find additional *MeCP2*-reactivating compounds, with the expectation that an *MeCP2*-reactivating compound will eventually be advanced to the clinic. Here we identified two additional molecules, AG490 and Jaki, that can reactivate *MeCP2* from Xi. Since both compounds inhibit the JAK/STAT pathway, we tested several additional molecules in the JAK/STAT inhibitor family, but we identified no additional Janus kinase inhibitors that could reactivate *MeCP2* (data not shown). Our data suggest that there is a preferential role for specific classes of Janus kinases in XCI maintenance or that these compounds are working through off-target effects. Alternatively, different dose regimens might be required to produce reactivating effects in a drug-dependent manner, suggesting that a large parameter space of dosing regimens must be explored to identify an effective and reliable *MeCP2*-reactivating protocol.

We expect time and cellular context to be particularly critical factors underlying the activity of these drugs, perhaps because of the temporal dynamics needed to produce DNA demethylation or long-term transcriptional changes [46, 47]. In support of this hypothesis, double treatments of AG490 had reactivating effects in primary cortical neurons, whereas single treatments did not. Furthermore, in the fibroblast clone Xi8, longer, double-dose treatments of AG490 produced significant downregulation of *Xist* and *Rnf12*, two key players of XCI [48], whereas the shorter/single administrations of AG490 did not (Suppl. Fig. 1B). Surprisingly, the double treatment (5-Aza + Janus kinase inhibitor) did not elicit a stronger MeCP2 reactivation, suggesting that AG490/Jaki-mediated MeCP2 reactivation could work through a mechanism that mechanistically overlaps with that of 5-Aza. We have also shown 10–20% MeCP2 reactivation in neurons at a single cell level (Fig. 2, Suppl. Fig. 1C) and modest MeCP2 reactivation by qRT-PCR and luminescence assays (Fig. 1, Suppl. Fig 1/2, 3B). The apparent discrepancies in measured reactivation levels are likely due to inherent methodological differences in the employed techniques (i.e., single vs population analysis).

While these data demonstrate the importance of temporal aspects when attempting to reactive *MeCP2* (discussed above), the cellular context is also an important variable. For example, while AG490 and 5-Aza reactivated MeCP2 in our mouse fibroblast cell lines, only 5-Aza treatments increased MeCp2 levels in the hamster/human chimeric cell line. These distinctive responses can also be linked to differences in XCI profiles between different cell lines and likely linked to *Xist* regulation [45] and suggest there may be species-specific differences (i.e., mouse vs human).

With the exception of the results in the chimeric line, which might not fully recapitulate the complexity of physiological XCI, our results suggest that AG490/JAKI may reactivate *MeCP2* by transcriptional downregulation of *Xist*. It is likely that neurons and other cell lines rely more robustly on *Xist*-mediated silencing than DNA methylation at regulatory regions. Indeed, it is clear that some cell lines are more dependent than others on *Xist* silencing, which may explain our observed differences between cell lines. For example, *Xist* RNA can be easily displaced from the Xi in cancer cells but not in primary cells [49]. Finally, while we did not extensively profile the reactivation of other X-linked genes by JAK-inhibitor treatment (other than Hprt), it is possible that these drugs can reactivate other X-linked genes such as *CDKL5* and *FMR1*. It remains to be addressed whether pretreatment levels of DNA methylation differ between cell lines and in their response to AG490. More studies are clearly needed to fully elucidate the mechanistic basis by which AG490 reactivates *MeCP2*, and whether similar effects can be observed in human neurons.

## Conclusions

In brief, our study shows that AG490 can reactivate the XCI-silenced copy of the *MeCP2* gene by in vitro and ex vivo approaches, and this general reactivation effect is observed across different types of cells (ex vivo neurons and fibroblasts) independently performed in three laboratories. Given the apparent safety of AG490 in neuronal culture treatments [50], our results indicate that AG490, and perhaps Jaki, could provide alternative approaches for reactivating *MeCP2* and potentially treating RTT. However, extensive medicinal chemistry is likely needed to identify more efficacious compounds, and rigorous studies are needed to test mechanism, safety, and efficacy in vivo.

## Supplementary Information

**Additional file 1:** **Supplementary figure 1.** *Compound validation under different treatment regimens*. **A**) AG490 validation in 6-well plate format. Relative Luminescence Units (RLU) is shown in function of the drug treatment for the clone Xi8. **B)** qRT-PCR of *MeCP2*, *luciferase*, *Xist* and *Rnf12*. Left: single treatment for the 72 hours regimen for clone Xi8. Right: double treatment regimen for 72 hr each for clone Xi8. Bars indicate standard error of the mean (SEM). * indicates statistical significance, *p* ≤ 0.05, n = 3 per genes, average of two independent assays is shown) (**C)** Top: Representative fluorescent immunostaining images of cortical neurons in presence or absence of the compounds with double treatment (i.e. twice for 72 hr). Bottom: Quantitative analysis of number of GFP positive cells. The % of GFP positive cells increased in the presence of AG490 and Jaki with a double treatment regimen. Bars indicate standard error of the mean (SEM), samples are color-coded, concentrations are shown. * indicates statistical significance, *p* ≤ 0.05, n = 4 per compound and dose, average of two independent assays is shown).

**Additional file 2:** **Supplementary figure 2.** 5*-Azacytidine and AG490 have no synergistic effect towards MeCP2 reactivation*. **A** and **B)** Single and double treatments for 5-Azacytidine (5-Aza) and AG490 in Xi8 clonal line are shown. **A**) Relative Luminescence Units (RLU) in function of the drug treatment is shown. Bars indicate standard error of the mean (SEM). * indicates statistical significance, *p* ≤ 0.05) (n = 3 per genes, average of two independent assays is shown). **B**) qRT-PCR of *MeCP2*, *luciferase*, and *Xist*. Double treatment regimen for 72 hr per treatments for clone Xi8 (see Materials and methods). Bars indicate standard error of the mean (SEM). * indicates statistical significance, *p* ≤ 0.05) (n = 3 per genes, average of two independent assays). DMSO: vehicle control 0.1%, 5-Aza [10 μM], AG490 [3 μM], 5-Aza + AG490: simultaneous treatment of two compounds [3 μM each], p(5-Aza) + AG490: p(5-Aza) indicated primed 5-Aza, meaning that priming by 5-Aza [3 μM] was administered before before AG490 [3 μM] treatment (see Materials and Methods). **C)** Drug treatments do not affect Xist localization on the inactive X chromosome in mouse cells. Xist RNA-FISH in C2C12, a female fully differentiated mouse female cell line. Drug treatment was performed for 4 days and drugs concentrations are shown. Xist, red; DNA (DAPI) in blue are shown. 2Xi per cell are visible in most cells as this cell line is mostly tetraploid [38].

**Additional file 3:** **Supplementary Table 1.** This table shows the libraries used in this screen.

**Additional file 4:** **Supplementary Table 2.** This tables shows tall the drugs used in this screen and the % of luciferase reactivation per drug (normalized values).

## Data Availability

All data generated or analyzed during this study are included in this published article and its supplementary information files.
