# Supplementary figures and images for: A small-molecule screen reveals novel modulators of MeCP2 and X-chromosome inactivation maintenance
Source: J Neurodev Disord. 2020 Nov 10;12:29. doi: 10.1186/s11689-020-09332-3 (PMC7657357; doi:10.1186/s11689-020-09332-3)

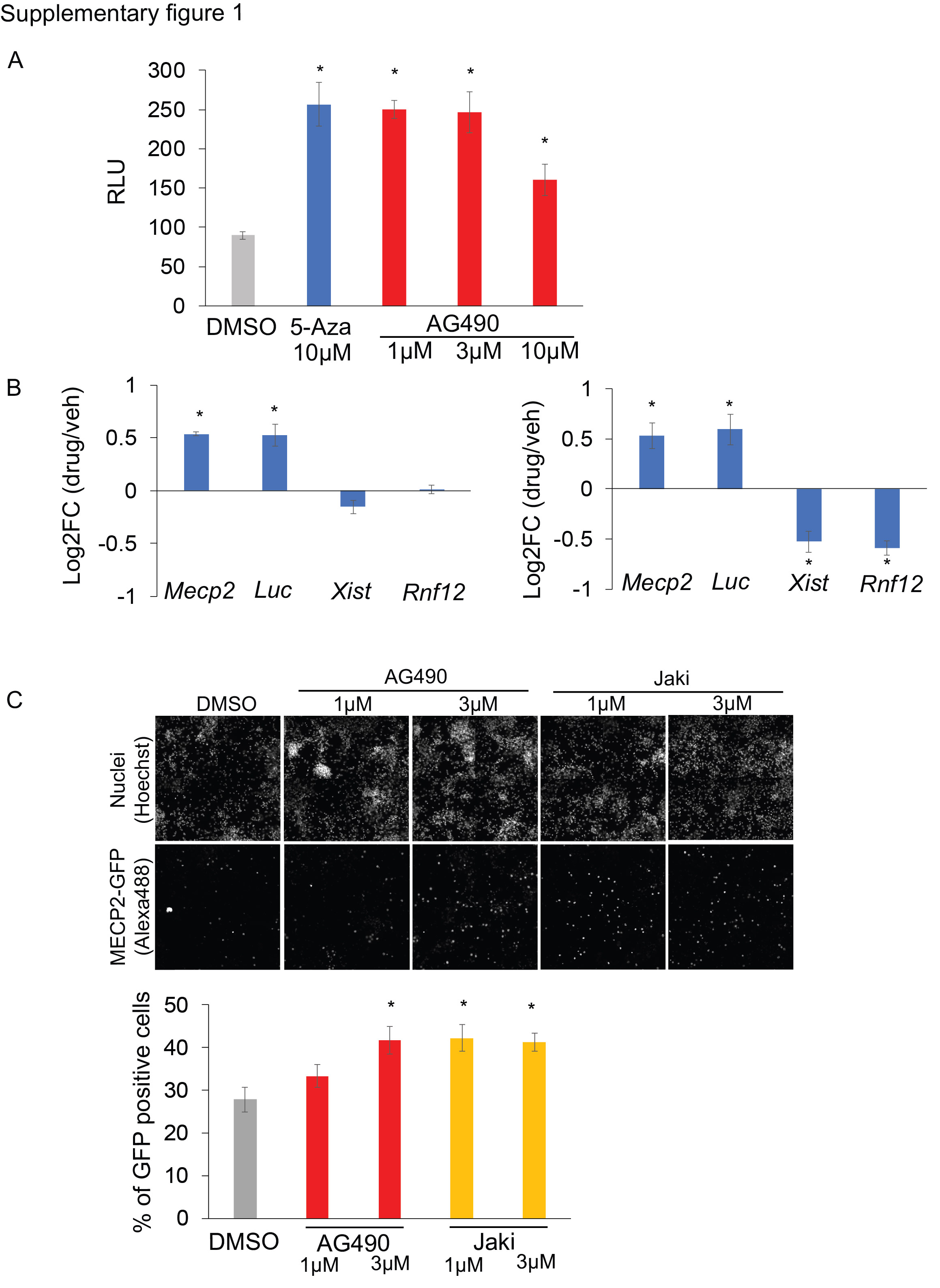

Supplement: Supplementary file 1 — Additional file 1: Supplementary figure 1. Compound validation under different treatment regimens. A) AG490 validation in 6-well plate format. Relative Luminescence Units (RLU) is shown in function of the drug treatment for the clone Xi8. B) qRT-PCR of MeCP2, luciferase, Xist and Rnf12. Left: single treatment for the 72 hours regimen for clone Xi8. Right: double treatment regimen for 72 hr each for clone Xi8. Bars indicate standard error of the mean (SEM). * indicates statistical significance, p ≤ 0.05, n = 3 per genes, average of two independent assays is shown) (C) Top: Representative fluorescent immunostaining images of cortical neurons in presence or absence of the compounds with double treatment (i.e. twice for 72 hr). Bottom: Quantitative analysis of number of GFP positive cells. The % of GFP positive cells increased in the presence of AG490 and Jaki with a double treatment regimen. Bars indicate standard error of the mean (SEM), samples are color-coded, concentrations are shown. * indicates statistical significance, p ≤ 0.05, n = 4 per compound and dose, average of two independent assays is shown). [file 11689_2020_9332_MOESM1_ESM.docx]

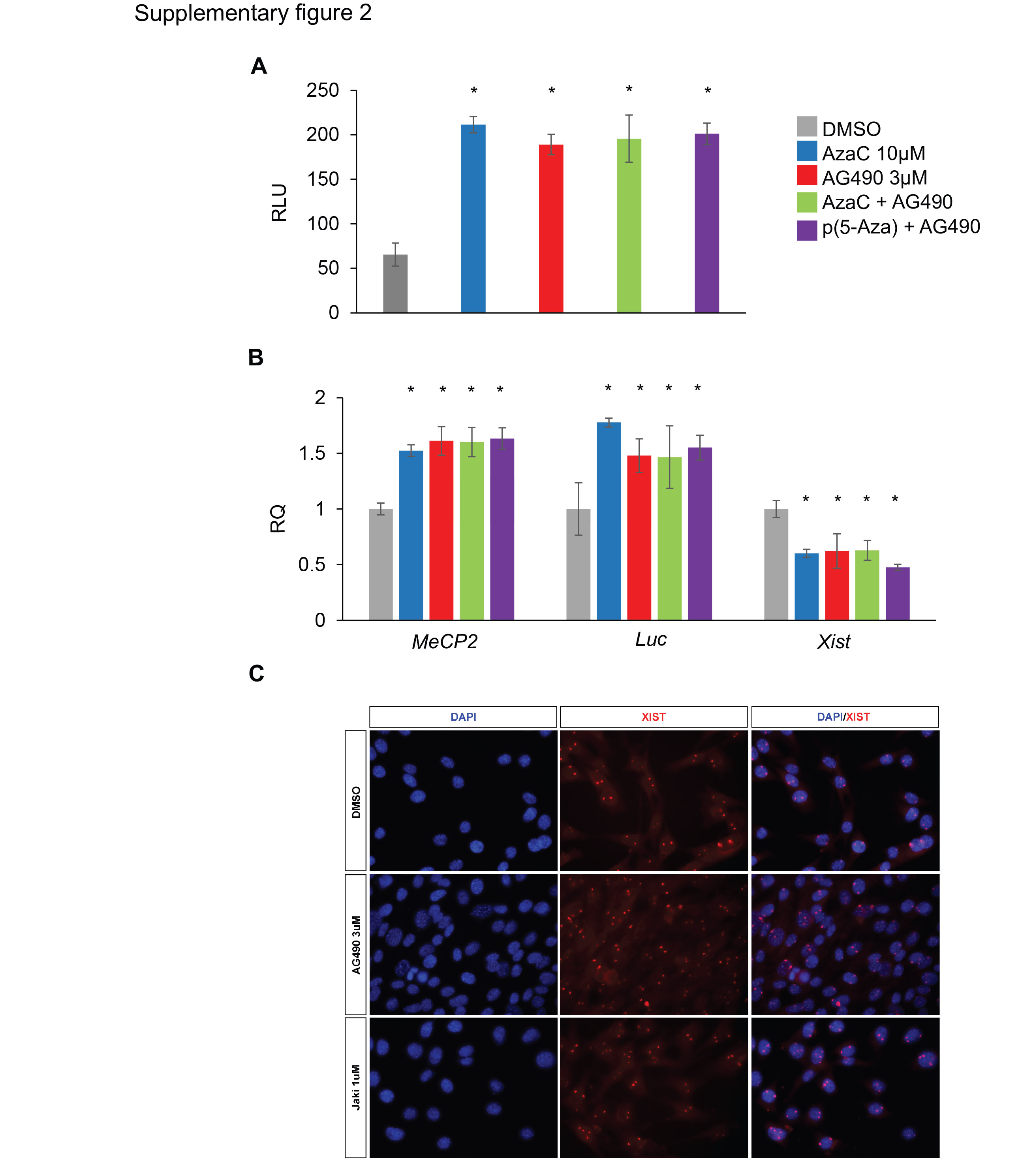

Supplement: Supplementary file 2 — Additional file 2: Supplementary figure 2. 5-Azacytidine and AG490 have no synergistic effect towards MeCP2 reactivation. A and B) Single and double treatments for 5-Azacytidine (5-Aza) and AG490 in Xi8 clonal line are shown. A) Relative Luminescence Units (RLU) in function of the drug treatment is shown. Bars indicate standard error of the mean (SEM). * indicates statistical significance, p ≤ 0.05) (n = 3 per genes, average of two independent assays is shown). B) qRT-PCR of MeCP2, luciferase, and Xist. Double treatment regimen for 72 hr per treatments for clone Xi8 (see Materials and methods). Bars indicate standard error of the mean (SEM). * indicates statistical significance, p ≤ 0.05) (n = 3 per genes, average of two independent assays). DMSO: vehicle control 0.1%, 5-Aza [10 μM], AG490 [3 μM], 5-Aza + AG490: simultaneous treatment of two compounds [3 μM each], p(5-Aza) + AG490: p(5-Aza) indicated primed 5-Aza, meaning that priming by 5-Aza [3 μM] was administered before before AG490 [3 μM] treatment (see Materials and Methods). C) Drug treatments do not affect Xist localization on the inactive X chromosome in mouse cells. Xist RNA-FISH in C2C12, a female fully differentiated mouse female cell line. Drug treatment was performed for 4 days and drugs concentrations are shown. Xist, red; DNA (DAPI) in blue are shown. 2Xi per cell are visible in most cells as this cell line is mostly tetraploid [38]. [file 11689_2020_9332_MOESM2_ESM.docx]
