# Supplementary material for: A small-molecule screen reveals novel modulators of MeCP2 and X-chromosome inactivation maintenance
Source: J Neurodev Disord. 2020 Nov 10;12:29. doi: 10.1186/s11689-020-09332-3 (PMC7657357; doi:10.1186/s11689-020-09332-3)
Supplement: Supplementary file 3 — Additional file 3: Supplementary Table 1. This table shows the libraries used in this screen. [file 11689_2020_9332_MOESM3_ESM.docx]

**Supplementary table 1**

| **Library** | **Class** | **Numbers** | **Hits** |
| --- | --- | --- | --- |
| SMART | CNS drugs | 320 |  |
| GSK KI | Kinase inhibitors | 637 |  |
| BDZ | benzodiazepine family | 14 |  |
| Tocris Mini | commercial / Tocris | 1120 |  |
| Prestwick | commercial / Prestwick | 1120 |  |
| X-901 | NIMH CNS drugs | 271 |  |
| NCC1 | NIH Clinical collection / most cancer drugs | 446 |  |
| NCC2 | NIH Clinical collection / most cancer drugs | 320 |  |
| Roth | Roth lab internal library / CNS and GPCR targeting | 456 |  |
| NCI diversity IV | NCI collection | 1596 |  |
| LOPAC | commercial / Sigma | 1280 |  |
| Spectrum | commercial / Microsource | 2400 |  |
| SL1700 | commercial / Selleckchem | 1836 |  |
| SL151 | commercial / Selleckchem | 151 | AG490 |
| Gallo extracts | natural products | 477 |  |
| UNC epigenetic collection | UNC CICBDD synthetic compounds | 1032 |  |
| UNC random collection | UNC CICBDD synthetic compounds | 15000 |  |
| Total # |  | 28,476 |  |
